# Revealing the Invisible: Circulating and Urinary Tumor DNA in Bladder Cancer

**DOI:** 10.3390/cancers18213566

**Published:** 2026-11-06

**Authors:** Sophia L. Qin, Benista Owusu-Amo, David T. Miyamoto

**Affiliations:** 1 Krantz Family Center for Cancer Research, Mass General Brigham Cancer Institute, Charlestown, MA 02129, USA; 2 Department of Radiation Oncology, Massachusetts General Hospital, Harvard Medical School, Boston, MA 02114, USA; 3 Broad Institute of MIT and Harvard, Cambridge, MA 02142, USA

**Keywords:** circulating tumor DNA, urinary tumor DNA, muscle-invasive bladder cancer, metastatic urothelial carcinoma, molecular residual disease, treatment selection, post-treatment surveillance, liquid biopsy, tumor-informed assays, tumor-naïve assays

## Abstract

Muscle-invasive bladder cancer (MIBC) carries a high risk of recurrence and metastatic progression despite aggressive treatment with radical cystectomy or trimodality therapy. Current surveillance methods, including imaging and cystoscopy, have limited sensitivity for detecting molecular residual disease (MRD) and early recurrence, highlighting the need for reliable biomarkers to guide treatment selection. Circulating tumor DNA (ctDNA) is a promising non-invasive biomarker that can provide real-time insights into tumor burden, treatment response, and recurrence risk. In this narrative review, we synthesize evidence on ctDNA’s potential in monitoring and guiding MIBC treatment. We discuss ctDNA detection methods and applications in genomic profiling, perioperative risk stratification, MRD detection, treatment selection, response monitoring, and post-treatment surveillance. Postoperative ctDNA positivity has been associated with significantly shorter disease-free survival (HR 6.93, 95% CI 2.40–20.05), and patients who failed to achieve ctDNA clearance during neoadjuvant chemotherapy had worse recurrence-free survival (HR 4.7, 95% CI 1.2–18.8). With further prospective validation, serial ctDNA monitoring may enable earlier detection of molecular recurrence and inform the timing and use of adjuvant therapy. The recent FDA approval of adjuvant atezolizumab for MIBC patients with ctDNA-detected MRD following cystectomy marks an important milestone in ctDNA-guided treatment selection. Given ctDNA’s limited sensitivity in detecting localized bladder recurrence, urinary tumor DNA (utDNA) may serve as a complementary biomarker to detect intravesical disease. Broader clinical implementation of these liquid biopsy strategies will require further validation, standardization, and efforts to reduce false-positive and false-negative results.

## Simple Summary

Bladder cancer can recur or metastasize after surgery, chemotherapy, and/or radiation. Current follow-up methods such as radiographic imaging, cystoscopy, and urine cytology may not detect residual cancer early enough to guide timely treatment. Liquid biopsies, such as circulating tumor DNA (ctDNA), cancer-derived DNA fragments found in the blood, are being investigated as tools for more precise monitoring of bladder cancer. In this review, we synthesize studies investigating whether ctDNA can identify patients at higher risk of recurrence, detect relapse earlier than standard imaging, and inform treatment decisions. We also discuss how urinary tumor DNA (utDNA) can complement ctDNA, as it detects residual cancer confined to the bladder with higher accuracy. Non-invasive liquid biopsy strategies, such as ctDNA and utDNA, have the potential to improve survival and quality of life for patients with bladder cancer by enabling earlier detection of recurrence and reducing unnecessary treatment.

## 1. Introduction

Bladder cancer represents a significant global health burden, ranking as the ninth most common cancer worldwide and the sixth most frequently diagnosed cancer in the United States [1,2]. In 2026, an estimated 84,530 new cases of bladder cancer will be diagnosed in the United States, resulting in 17,870 deaths [3]. The most common type of bladder cancer is urothelial carcinoma, which accounts for over 90% of cases. Among patients diagnosed with urothelial carcinoma, approximately 25% of patients have muscle-invasive bladder cancer (MIBC) [4], which is characterized by the cancerous invasion of the muscularis propria or beyond (≥T2 disease) [5]. Despite recent therapeutic advances, clinical outcomes in MIBC remain poor, with a 5-year overall survival (OS) rate of approximately 50% [6]. Approximately half of patients with MIBC develop metastatic urothelial carcinoma (mUC) within 2 to 3 years of diagnosis [7,8].

Due to its high propensity for metastasis, the longstanding management of MIBC has consisted of aggressive multimodal treatment with radical cystectomy (RC) and systemic chemotherapy [9]. For patients undergoing RC, perioperative systemic therapy is individualized according to cisplatin eligibility and pathological risk. Cisplatin-eligible patients may receive neoadjuvant cisplatin-based chemotherapy [10]. Perioperative options also include neoadjuvant cisplatin-based chemotherapy with immune checkpoint inhibition and antibody–drug conjugate–immune checkpoint inhibitor combinations for appropriate patients [11]. Postoperative pathological risk, including pT3/4 and/or pN+ disease, may further guide adjuvant chemotherapy or immunotherapy [12].

As an alternative to RC, selected patients with localized MIBC may undergo bladder-sparing trimodality therapy (TMT), consisting of maximal transurethral resection of bladder tumor (TURBT) followed by concurrent chemoradiotherapy (CRT) [13]. Long-term retrospective studies have shown that TMT yields OS and disease-specific survival (DSS) outcomes comparable to RC in select patients [13]. TMT reduces the risk of surgical complications and preserves native bladder function, helping maintain patients’ quality of life [13,14,15,16]. Nevertheless, 31.8% of patients treated with TMT and 41.3% undergoing RC experience distant recurrence [17], underscoring the need for biomarkers that detect residual disease, improve risk stratification, and inform treatment selection.

Currently, bladder cancer management and surveillance primarily rely on clinicopathological assessments, including cross-sectional imaging, cystoscopy, and urine cytology. However, these methods have important limitations for detecting molecular residual disease (MRD), microscopic disease that persists after curative-intent treatment [18]. CT and MRI generally identify recurrence only after sufficient tumor burden becomes radiographically apparent and therefore often cannot detect MRD [19]. Additionally, cystoscopy provides direct visualization of the bladder but is limited to assessing intravesical disease and cannot detect occult extravesical or systemic recurrence [4]. Urine cytology sensitivity varies with both tumor grade and stage and is particularly limited for low-grade and earlier-stage disease. In one prospective study, sensitivity was 16% for low-grade tumors compared with 84% for high-grade tumors, and higher tumor stage was independently associated with improved cytologic sensitivity [20]. Cytology sensitivity is also influenced by specimen cellularity and collection method. In voided urine, 20–39 urothelial cells per 10 high-power fields represented the optimal cellularity range, with sensitivity declining at both lower and higher cellularity. Instrumented specimens demonstrated higher sensitivity than voided specimens (82% vs. 25%) [21]. Together, these limitations highlight the need for more sensitive biomarkers for earlier disease detection.

Circulating tumor DNA (ctDNA) analysis offers a non-invasive adjunct to conventional surveillance methods with several distinct advantages. Cell-free DNA (cfDNA) refers to extracellular DNA released from cells throughout the body into the blood. ctDNA is the tumor-derived fraction of cfDNA. While most cfDNA originates from normal cells, tumor cells release ctDNA into the bloodstream through apoptosis, necrosis, and active secretion [22]. ctDNA can complement classical tissue biopsy by revealing tumor heterogeneity and longitudinal tumor-derived alterations that may not be represented in a single-site tissue sample [22]. This is particularly valuable for metastatic bladder cancer, where the genomic landscape of the primary tumor differs from that of metastatic lesions [23]. Additionally, ctDNA can capture dynamic tumor changes because it has a relatively short half-life of less than 2 h [24]. As a result, ctDNA can reflect tumor status at a given time point, with higher ctDNA levels associated with greater tumor burden, progression, or recurrence [25]. Therefore, plasma ctDNA monitoring may prove to be a beneficial adjunct to standard approaches such as physical exams, cross-sectional imaging, cystoscopy, and urine cytology [26].

Growing evidence supports the prognostic validity of ctDNA in MIBC, while evidence of predictive validity and clinical utility is emerging in selected adjuvant settings. Studies have evaluated ctDNA for genomic profiling, risk stratification, treatment selection, and post-treatment surveillance [18,27,28]. This review focuses predominantly on MIBC because these patients remain at substantial risk of occult systemic disease and metastatic recurrence despite curative-intent treatment [17]. In contrast, evidence supporting plasma ctDNA monitoring in NMIBC remains limited, in part because bladder-confined tumors may shed insufficient tumor DNA into the circulation for reliable detection [29]. Accordingly, our discussion of NMIBC focuses primarily on urinary tumor DNA (utDNA), which may be better suited to detecting localized intravesical disease [29,30,31,32].

## 2. Methods

We identified the literature for this narrative review by searching PubMed and Google Scholar, supplemented by manually reviewing reference lists from relevant publications. We searched ClinicalTrials.gov for ongoing clinical trials and consulted U.S. Food and Drug Administration (FDA) sources for current regulatory approvals. While most references were full-text publications, we included certain conference abstracts when they reported clinically relevant findings. We last updated the literature search on 9 October 2026. All included publications were in English and were published between 2008 and 2026. We did not apply a prespecified lower publication-date cutoff; we included earlier studies within this range when relevant to foundational aspects of ctDNA biology and detection. Search terms included combinations of “bladder cancer,” “muscle-invasive bladder cancer,” “metastatic urothelial carcinoma,” “circulating tumor DNA,” “ctDNA,” “cell-free DNA,” “molecular residual disease,” “minimal residual disease,” “MRD,” “liquid biopsy,” “urinary tumor DNA,” “utDNA,” “radical cystectomy,” “trimodality therapy,” “neoadjuvant chemotherapy,” “immunotherapy,” “adjuvant therapy,” “treatment response,” “recurrence,” and “surveillance.” Eligible studies primarily included clinical studies of ctDNA in MIBC evaluating genomic profiling, risk stratification, MRD detection, treatment response, treatment selection, or surveillance. We included selected studies of metastatic urothelial carcinoma when they provided evidence regarding ctDNA assay performance or genomic profiling relevant to MIBC. We also selectively included NMIBC studies when they addressed limitations of plasma ctDNA or the complementary role of utDNA. We excluded studies unrelated to urothelial carcinoma, ctDNA, or related liquid biopsy biomarkers. We discuss ctDNA and utDNA assays and analytical platforms based on their use in clinically relevant bladder cancer studies and to illustrate distinct methodological approaches. These examples are illustrative and do not represent an exhaustive list of available assays or platforms.

We selected studies for Table 1 based on their relevance to the prognostic or predictive validity of ctDNA in MRD detection, treatment-response assessment, surveillance, or treatment selection. Because this is a narrative review, we synthesized findings qualitatively by clinical application, without formal meta-analysis or risk-of-bias assessment.

## 3. ctDNA Detection Technologies and Approaches

The tumor-derived portion of cfDNA, ctDNA, provides information about disease burden. ctDNA has been shown to correlate with serum alkaline phosphatase and lactate dehydrogenase levels, both well-established metrics of tumor volume [33]. In metastatic bladder cancer, ctDNA can comprise up to 50% of circulating cfDNA, whereas in localized muscle-invasive disease, it typically represents a much smaller fraction (1% or less) [34]. Because ctDNA comprises a small fraction of total cfDNA, especially in MIBC, highly sensitive detection methods are required. ctDNA can be distinguished from non-tumor cfDNA by identifying tumor-specific genomic alterations, ranging from large-scale chromosomal changes to individual somatic mutations [35]. In addition, ctDNA fragments tend to be shorter and exhibit greater variability in length than cfDNA originating from non-cancer cells [36]. ctDNA also exhibits characteristic fragmentation patterns generated by nucleases, including specific end motifs, preferred cleavage sites, and jagged ends [36,37,38]. Lastly, ctDNA can carry tumor-associated epigenetic alterations, including hypermethylation at selected CpG sites or gene promoters [39]. Modern ctDNA detection assays use analytically distinct genetic, fragmentomic, or epigenetic approaches to distinguish ctDNA from non-tumor cfDNA [40].

**Figure 1:**
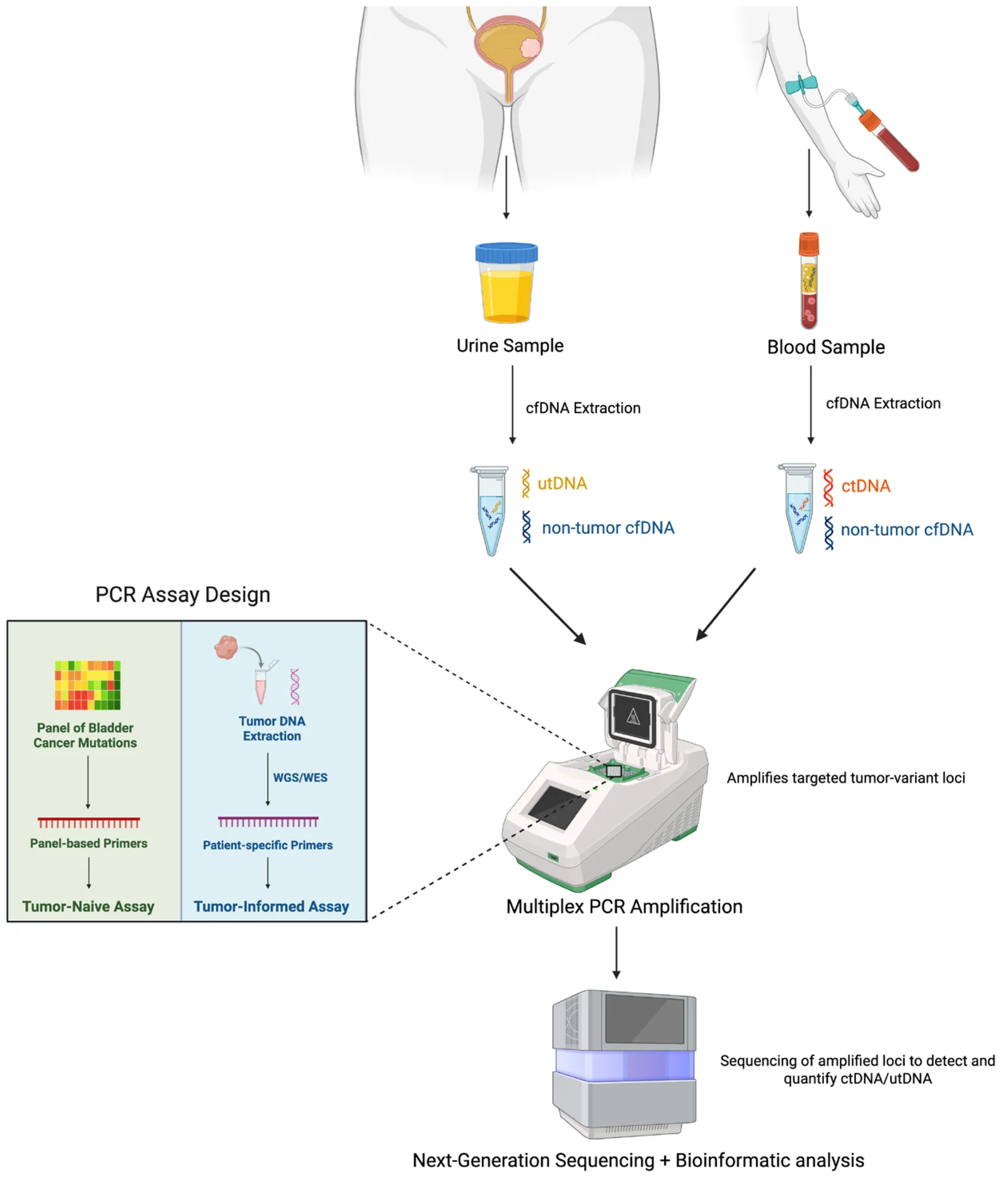
Tumor-informed and tumor-naïve detection of ctDNA and utDNA in MIBC. Cell-free DNA can be extracted from urine or plasma to detect urinary tumor DNA (utDNA) or circulating tumor DNA (ctDNA), respectively. Tumor-naïve assays use predefined panels of common bladder cancer mutations to target fixed genomic loci. In contrast, tumor-informed assays sequence patient-derived tumor tissue (e.g., WGS or WES) to identify patient-specific somatic variants and design personalized primers. Targeted loci are then amplified by multiplex PCR, sequenced by NGS, and analyzed bioinformatically to detect and quantify ctDNA and utDNA.

Among mutation-based ctDNA detection approaches, assays can be categorized as tumor-informed or tumor-naïve (Figure 1). Tumor-informed assays first sequence patient-derived tumor tissue, often with matched normal DNA, to identify tumor-specific somatic variants. These variants are then used to design a personalized multiplex PCR assay, which amplifies targeted loci from plasma cfDNA [41]. Next-generation sequencing (NGS) then analyzes the amplified cfDNA to detect and quantify ctDNA. In contrast, tumor-naïve assays do not require prior tumor tissue sequencing. Instead, they analyze plasma cfDNA using fixed panels of common cancer-specific mutations, with bioinformatic filtering to identify tumor-derived variants [41].

Because tumor-informed assays generally provide greater sensitivity for MRD detection, most ctDNA studies in bladder cancer have used tumor-informed methods, particularly the Signatera™ MRD assay (Natera, Inc., San Carlos, CA, USA) [42]. After several years under Breakthrough Device Designation, Signatera™ CDx received FDA approval in May 2026 as a companion diagnostic for identifying ctDNA MRD-positive patients with MIBC who may benefit from adjuvant atezolizumab [43]. Most published data to date have used the whole-exome sequencing (WES)-based Signatera™ assay, which detects up to 16 patient-tumor-specific somatic single-nucleotide variants (SNVs) for longitudinal ctDNA monitoring. Subsequent plasma samples can be analyzed, enabling real-time assessment of disease status and treatment response. A sample is considered ctDNA-positive when at least two tracked variants are detected. In addition to distinguishing ctDNA-positive versus negative samples, the Signatera™ assay quantifies ctDNA burden as the mean number of tumor molecules per mL of plasma (MTM/mL) [44]. In 2025, Natera launched Signatera™ Genome, a whole-genome sequencing (WGS)-based version of its tumor-informed MRD assay that expanded the personalized panel from 16 to 64 clonal tumor-specific variants [45,46]. Compared with the WES-based assay, Signatera™ Genome demonstrated a lower analytical limit of detection and a median lead time of three months in detecting relapse [46]. These methods enable sensitive detection and analysis of ctDNA despite its low abundance, allowing non-invasive characterization of the tumor genetic landscape through plasma samples.

## 4. ctDNA for Genomic Profiling

ctDNA-based gene mutation detection can non-invasively reconstruct the tumor tissue driver genome with high accuracy, providing clinically actionable insight that may guide targeted treatment selection. In a cohort of 51 patients with either MIBC or metastatic bladder cancer, Vandekerkhove et al. performed WES and targeted sequencing of 50 bladder cancer driver genes in plasma cfDNA, identifying recurrent alterations in clinically relevant pathways across all ctDNA-positive patients [27]. Frequent alterations were observed in tumor suppressor genes such as *TP53* and *RB1*, oncogenes including *MDM2*, and chromatin-modifying genes such as *ARID1A*. Additionally, ctDNA profiling identified potentially targetable alterations in the MAPK/ERK and PI3K/AKT/mTOR signaling pathways, including *ERBB2* amplification, *PIK3CA* hotspot mutations, and a novel *FGFR3* fusion [27]. Identifying these alterations through ctDNA may inform the use of targeted therapies, such as AKT inhibitors for patients with PI3K/AKT/mTOR pathway aberrations [47]. Furthermore, ctDNA profiling identified mutations in DNA damage repair (DDR) genes such as *ERCC2* and *ATM* [27]. *ATM* alterations have been associated with improved responses to cisplatin-based neoadjuvant chemotherapy [48], and *ERCC2* mutations with favorable long-term outcomes following chemoradiation [49]. These findings show that ctDNA can detect tumor-specific alterations that may guide targeted interventions.

For ctDNA to serve as a reliable tool for molecular profiling, it must accurately represent the underlying tumor genome. To evaluate this, Vandekerkhove et al. compared ctDNA from 104 patients with mUC to matched tumor tissue samples obtained during cystectomy [23]. Among patients with matched tumor tissue and plasma samples, they compared the plasma sample with the highest ctDNA fraction with the most recent tumor tissue specimen. Targeted sequencing identified 265 somatic coding mutations, of which 83.4% were detected in both ctDNA and matched tumor tissue. Additionally, seven known cancer-related genes (*ERBB2*, *ERBB3*, *PPARG*, *MYC*, *CCND1*, *CCNE1*, and *MDM2*) were amplified in both ctDNA and tumor tissue samples [23]. This high concordance suggests that ctDNA-based liquid biopsies may complement tissue biopsies when tissue availability is limited. This study also found that bulk metastatic driver alterations in serial ctDNA remained relatively consistent over time, while serial primary tumor biopsies exhibited greater temporal heterogeneity [23]. Tissue biopsy provides a snapshot of a lesion at a single time point, whereas ctDNA reflects tumor-derived DNA shed from multiple sites; together, they offer a more comprehensive view of the tumor’s genomic landscape.

## 5. Pre-Treatment ctDNA for Risk Stratification

ctDNA is a highly sensitive, non-invasive biomarker for personalized risk assessment in the perioperative management of MIBC. Alongside current risk stratification methods, such as imaging, histopathological grading, and staging [50], ctDNA analysis may enhance risk stratification by detecting microscopic disease changes in real time [28,50].

Pre-operative ctDNA detection before RC is strongly associated with higher recurrence risk and poorer survival. Christensen et al. conducted a prospective, nonrandomized longitudinal study evaluating ctDNA throughout treatment in patients with urothelial bladder carcinoma [28]. ctDNA positivity before neoadjuvant chemotherapy (NAC) and before RC identified patients at increased risk of recurrence. In contrast, ctDNA-negative patients rarely recurred during the 12-month monitoring period, suggesting that preoperative ctDNA status can help distinguish patients with different prognoses. Specifically, recurrence occurred in 75% of ctDNA-positive patients compared with only 7% of ctDNA-negative patients [28]. Sfakianos et al. further supported these findings in a retrospective study, which found that ctDNA status was the only risk factor independently associated with shorter disease-free survival (DFS) in multivariable analyses that accounted for sex, age, neoadjuvant therapy status, and tumor mutational burden [51].

ctDNA status also correlates with pathological outcomes following neoadjuvant therapy. In the Christensen cohort, all patients who were ctDNA-positive before RC had residual disease (≥T1) and/or lymph node metastases at cystectomy, while all patients with pT0 were ctDNA-negative [28]. The single-arm phase II ABACUS trial found that MIBC patients who were ctDNA-negative at baseline or after neoadjuvant atezolizumab were more likely to achieve pCR [52]. Associations of pretreatment ctDNA status with recurrence and pathologic response have prompted investigation into whether ctDNA can guide treatment selection, including response-adapted bladder-preservation strategies [53].

## 6. ctDNA for Post-Treatment Surveillance and MRD Detection

The most extensively studied application of ctDNA in MIBC is detecting MRD after RC. Despite undergoing RC, many patients harbor occult micrometastases that ultimately lead to recurrence [54]. ctDNA can detect MRD before radiographic recurrence becomes apparent. In MIBC, ctDNA detection precedes radiographic recurrence by a median of 6 months (*p* = 0.024), providing a window for earlier intervention and risk-adapted postoperative management [55].

In a prospective longitudinal cohort study, Christensen et al. found that postoperative ctDNA positivity indicated MRD and that serial ctDNA measurements could detect recurrence during surveillance. Following RC, serial ctDNA analysis identified all 13 patients who subsequently developed metastatic relapse (100% sensitivity), whereas no recurrences occurred among the 47 patients who remained ctDNA-negative during surveillance [28]. Despite these striking results, the relatively small number of recurrence events must be considered when interpreting these estimates. In this cohort, postoperative ctDNA status was more strongly associated with recurrence-free survival than conventional clinicopathologic factors, including lymph node status and pathologic downstaging [28]. ctDNA also preceded radiographic detection of recurrence by a median of 96 days (*p* = 0.021) [28]. These findings provided some of the earliest evidence that ctDNA could serve as a reliable biomarker of MRD, enabling both recurrence risk stratification and earlier detection of relapse than conventional surveillance methods.

While postoperative ctDNA positivity identifies patients at high risk of recurrence, another important question is whether persistently ctDNA-negative patients require the same intensive surveillance protocols. Current surveillance strategies rely on serial CT, MRI, and increasingly PET-CT imaging to detect recurrence. However, repeated surveillance imaging may increase financial burden and cumulative radiation exposure, particularly among younger patients requiring long-term follow-up [56,57]. As a result, investigators have explored whether ctDNA could guide the intensity of postoperative surveillance. A study by Eraky et al. evaluated whether ctDNA could be incorporated into postoperative surveillance protocols to better identify patients at low risk of recurrence. By collecting postoperative CT/MRI/PET-CT scans of the chest, abdomen, and pelvis every 3 to 6 months and serial ctDNA samples every 6 to 12 weeks, they found an 82.6% concordance between ctDNA and imaging [56]. Among patients with discordant findings, most with detectable ctDNA but no radiographic evidence of recurrence later relapsed, suggesting that ctDNA may detect recurrence before it becomes visible on imaging [56]. Overall, ctDNA demonstrated a sensitivity and negative predictive value of 100% (95% CI, 100–100% for both), suggesting that persistently undetectable ctDNA may identify patients at low risk of recurrence during a median follow-up of 16 months [56]. However, these performance estimates require validation in larger, prospective surveillance cohorts before ctDNA can be used to safely reduce imaging intensity. If validated, incorporating ctDNA into standard surveillance protocols may enable earlier relapse detection while allowing less frequent imaging for patients with persistently undetectable ctDNA, thereby reducing radiation exposure and the burden of long-term postoperative monitoring.

While most studies have focused on ctDNA surveillance following radical cystectomy, there is a growing interest in its role for patients undergoing bladder-preserving approaches [14]. Recent studies have investigated the association between ctDNA and recurrence following TMT. Epstein et al. analyzed serial plasma ctDNA measurements alongside cross-sectional imaging and cystoscopic surveillance to characterize recurrence patterns [58]. Both pre-CRT and post-CRT ctDNA positivity were strongly associated with metastatic recurrence, with the first post-CRT assessment showing the strongest prognostic association (*p* < 0.0001) [58]. Furthermore, ctDNA detection after TMT accurately identified all patients who subsequently developed metastatic disease and preceded clinical detection of metastasis [58,59]. However, several patients experienced localized bladder recurrence without detectable ctDNA, suggesting that post-TMT ctDNA may be more sensitive for detecting metastatic relapse than local recurrence [58]. This limitation may reflect the relatively low shedding of ctDNA from tumors confined to the bladder [60,61].

## 7. ctDNA-Guided Adjuvant Therapy

Given the high recurrence rate of bladder cancer after RC, adjuvant immunotherapy has been evaluated for its potential to improve outcomes in patients with MIBC. A prospective, exploratory ctDNA analysis of the randomized phase III IMvigor010 trial provided some of the first evidence that ctDNA may have predictive utility for adjuvant immunotherapy [62]. IMvigor010 enrolled patients with MIBC after RC or nephroureterectomy with lymph node dissection and assigned them to either adjuvant atezolizumab or observation [63]. Among patients who were ctDNA-positive after surgery, those treated with atezolizumab had improved disease-free survival (DFS) and overall survival (OS) compared with those who underwent observation. ctDNA clearance occurred more frequently in the atezolizumab arm, suggesting that adjuvant immunotherapy may promote MRD eradication. In contrast, for ctDNA-negative patients, DFS and OS did not differ between the atezolizumab and observation arms [62], suggesting limited benefit from adjuvant immunotherapy in this subgroup. Further tumor-tissue transcriptional analyses identified molecular features associated with response to adjuvant atezolizumab among ctDNA-positive patients. Non-relapse was associated with immune-response and basal-squamous signatures, whereas relapse was associated with angiogenesis and fibroblast TGFβ signatures [62]. Together, these findings provided exploratory evidence that ctDNA may predict which patients are more likely to benefit from adjuvant atezolizumab. However, because treatment was not assigned according to ctDNA status [63], IMvigor010 did not establish that ctDNA-guided treatment directly improves clinical outcomes.

Following IMvigor010, Powles et al. conducted IMvigor011, a prospective randomized phase III trial designed to validate ctDNA-guided adjuvant atezolizumab in MIBC [18]. IMvigor011 prospectively monitored patients with no radiographic evidence of disease after RC using serial ctDNA testing for up to 1 year. Patients who became ctDNA-positive during surveillance were randomly assigned to atezolizumab or placebo, whereas persistently ctDNA-negative patients were observed without treatment. Atezolizumab improved outcomes among ctDNA-positive patients, increasing median DFS from 4.8 to 9.9 months and median OS from 21.1 to 32.8 months compared with placebo [18]. Rather than supporting uniform adjuvant treatment for all patients after cystectomy, IMvigor011 showed that patients with ctDNA-detectable MRD benefit from adjuvant atezolizumab. This ctDNA-guided approach has now led to FDA approval of adjuvant atezolizumab for patients with ctDNA-detectable MRD after cystectomy [43].

The TOMBOLA trial, a prospective, nonrandomized phase II study, investigated whether serial ctDNA monitoring could further refine ctDNA-guided adjuvant therapy by adapting treatment decisions to changes in disease status over time [64]. TOMBOLA prospectively monitored ctDNA in both high- and low-risk MIBC patients after NAC and RC, and treated those who tested ctDNA-positive with atezolizumab [65]. Conversely, patients who remained ctDNA-negative did not receive immunotherapy unless radiographic evidence of recurrence emerged [65]. By using ctDNA to guide treatment escalation or de-escalation, TOMBOLA sought to individualize adjuvant treatment using ctDNA-detected MRD rather than clinicopathologic risk factors alone. At 1 year after RC, RFS was 97% in ctDNA-negative versus 76% in ctDNA-positive patients (HR 8.7, 95% CI 2.7–28.2; *p* < 0.0001) [65]. TOMBOLA found that among high-risk patients, ctDNA positivity was associated with significantly worse RFS, while the difference within the low-risk subgroup did not reach statistical significance, suggesting that ctDNA can further refine risk stratification [65]. Previous use of adjuvant atezolizumab was restricted to patients classified as high-risk based on pathology and clinical assessment, which may have resulted in both undertreatment and overtreatment. Only half of ctDNA-positive patients met conventional high-risk criteria, whereas 30% of ctDNA-negative patients were classified as high risk, suggesting that pathology alone may both overlook patients with MRD and expose others to unnecessary treatment [65].

TOMBOLA’s nonrandomized, single-arm design limits causal conclusions regarding the clinical benefit of the ctDNA-guided strategy itself [65]. However, together with the randomized IMvigor trials, TOMBOLA strengthens the rationale for serial ctDNA monitoring to refine postoperative risk stratification and treatment adaptation based on MRD status [18,62,63,65].

## 8. utDNA as a Complement to ctDNA

Several studies have provided robust evidence that ctDNA can identify MRD, predict recurrence risk, and detect relapse earlier than imaging. However, studies investigating ctDNA in the post-TMT setting suggest that ctDNA is less sensitive for tumors confined to the bladder [58].

In the integrated RETAIN-1/2 analysis, 52% of MIBC patients developed local recurrence, but fewer than 10% were ctDNA-positive [66]. These findings suggest that plasma ctDNA alone does not reliably reflect residual intravesical disease, highlighting the need for additional biomarkers to monitor local disease status, such as urinary tumor DNA (utDNA) [58]. utDNA refers to tumor-derived DNA fragments shed directly into urine [67]. Because bladder tumors are in direct contact with the urinary tract, localized disease may release sufficient utDNA for detection but relatively little ctDNA into the systemic circulation [58,67]. Consequently, plasma ctDNA levels in bladder-confined disease may fall near or below current assay detection limits. At low ctDNA fractions, technical factors, including sequencing depth and the number of tumor-specific variants analyzed, may further limit detection [23]. Reduced plasma ctDNA sensitivity for bladder-confined disease supports utDNA as a complementary biomarker for monitoring localized recurrence and stratifying risk.

Growing clinical interest in utDNA has driven the development of increasingly sensitive platforms to detect tumor-derived DNA in urine. High-sensitivity utDNA detection methods include next-generation sequencing (NGS), digital droplet PCR (ddPCR), and methylation-based assays [68]. Practical requirements vary across platforms. Among NGS-based approaches, UroAMP is one of the most extensively evaluated utDNA assays. In its technical validation, UroAMP used 4-mL aliquots of preserved voided urine for targeted deep sequencing of 60 urothelial cancer–associated genes and low-pass whole-genome sequencing to detect mutations, copy-number alterations, loss of heterozygosity, and aneuploidy [69,70]. The assay demonstrated 97.4% sensitivity for variants at 1% variant allele frequency, supporting its ability to detect low-abundance tumor-derived alterations in urine [69,70]. ddPCR is a more targeted approach that interrogates predefined variants. For ddPCR-based urinary TERT mutation detection, the limit of detection can be as low as 0.18% mutant allele fraction using 40 ng DNA [71]. Methylation-based assays interrogate epigenetic rather than sequence alterations and have distinct preprocessing and DNA-input requirements. The utMeMA-based assay used 100 ng of pellet-derived DNA from 50 to 100 mL of voided urine, followed by bisulfite conversion [72].

Another well-characterized approach is urine Cancer Personalized Profiling by Deep Sequencing (uCAPP-Seq), a high-throughput sequencing (HTS) platform developed by Dudley et al. [73]. For uCAPP-Seq, a median of 50 mL of voided urine was centrifuged, and cfDNA was extracted from the supernatant using a resin-based method. The assay used a custom targeted panel spanning approximately 311 kb across 460 bladder cancer–relevant genes, and libraries were sequenced on an Illumina HiSeq 4000 (Illumina, Inc., San Diego, CA, USA). Using this approach, the median urinary cfDNA concentration was 7.7 ng/mL, with a median total yield of 348.1 ng per sample [73]. When uCAPP-Seq was evaluated in patients with early-stage bladder cancer, a median of 66.7% of mutations detected in tumor tissue were also identified in corresponding utDNA samples, while a median of 73.2% of mutations detected in utDNA were confirmed in paired tumor tissue, demonstrating strong concordance. Using uCAPP-Seq, tumor-naïve utDNA profiling detected localized bladder cancer with 83–84% sensitivity, while tumor-informed profiling achieved 93% sensitivity [73].

Advances in utDNA sequencing technologies have substantially improved the detection of localized bladder cancer, yet the field effect remains an important consideration because it can reduce utDNA specificity [74]. The field effect refers to somatic mutations in tumor-adjacent normal urothelial cells that can shed DNA into the urine, confounding the distinction between true residual disease and background mutations. To address the field effect, Shi et al. developed RePhyNERX, a method that filters out non-tumor-specific variants identified in adjacent urothelium and post-resection urine [74]. Incorporation of RePhyNERX into uCAPP-Seq significantly improved the prognostic performance of utDNA. In a prospective cohort of NMIBC patients treated with TURBT and BCG, RePhyNERX-enhanced uCAPP-Seq showed that post-BCG utDNA status was strongly associated with recurrence (HR 12.8, *p* < 0.001). utDNA status also enabled accurate classification of patients into molecular response categories: surgical responders, BCG responders, and non-responders [74].

Beyond the field effect, urologic instrumentation may also influence urinary DNA assay performance. In a prospective study using a cell-based urine DNA mutation and methylation assay, Dahmcke et al. found lower specificity in samples collected immediately after flexible cystoscopy than in those collected before cystoscopy (81.7% vs. 87.5%, *p* = 0.04) [75]. Preanalytical handling is also important because urinary cfDNA is highly unstable. Eberhard et al. showed that urinary cfDNA degrades rapidly in unstabilized samples, whereas preservation with urine-specific stabilization reagents maintained cfDNA concentration and fragment integrity during storage [76]. Additionally, urinary tract inflammation may alter the background composition of urinary cfDNA through increased shedding of immune-cell-derived DNA. In patients with bacterial urinary tract infection, Burnham et al. found that increasing urinary leukocyte counts led to a lower proportion of tissue-specific cfDNA, suggesting that inflammatory cell influx can dilute disease-specific DNA signals in urine [77]. Standardizing preanalytical collection and handling while accounting for clinical confounders such as inflammation may improve the reliability of utDNA analysis.

In a retrospective, non-randomized pilot study, Birkenkamp-Demtröder et al. compared ctDNA and utDNA detection in patients with non-muscle-invasive bladder cancer (NMIBC) [29], defined as disease confined to the urothelium or lamina propria without invasion of the muscularis propria [78]. Among 12 NMIBC patients, utDNA was detected in all patients who experienced disease progression, whereas ctDNA was detected in only 67% of these cases [29]. These findings support the potential prognostic value of utDNA for localized bladder cancer. In the single-arm phase II SWOG S1605 trial, St. Laurent et al. evaluated whether utDNA profiling could stratify the risk of treatment failure in patients with BCG-unresponsive NMIBC treated with atezolizumab. Baseline utDNA positivity was associated with lower complete response rates and poorer event-free survival, while detectable utDNA after 3 months of atezolizumab treatment was associated with subsequent treatment failure, even among patients with no clinical evidence of disease [30].

The clinical utility of utDNA has not yet been established and is being evaluated prospectively in ongoing trials. For high-risk NMIBC patients, the randomized TRUCE-LB01 trial is investigating whether utDNA can guide repeat TURBT (re-TURBT) by identifying patients with MRD who may benefit from additional tumor resection. This trial will evaluate whether NMIBC patients who are utDNA-negative may be spared from this unnecessary invasive procedure without compromising oncologic safety. Thus, TRUCE-LB01 is investigating utDNA as a potential tool to reduce overtreatment and enable more individualized management of high-risk NMIBC [31]. More recently, Qie et al. evaluated utDNA-guided treatment de-escalation in patients with very-high-risk NMIBC who achieved a complete response to systemic therapy. Among 21 utDNA-negative patients who underwent early immunotherapy cessation, RFS (HR 1.37, 95% CI 0.21–8.87, *p* = 0.73), OS (HR 2.48, 95% CI 0.12–53.7, *p* = 0.51), and duration of response (HR 1.33, 95% CI 0.21–8.50, *p* = 0.76) did not differ significantly from those receiving longer treatment. However, the nonrandomized design and small cohort require prospective validation [32].

**Figure 2:**
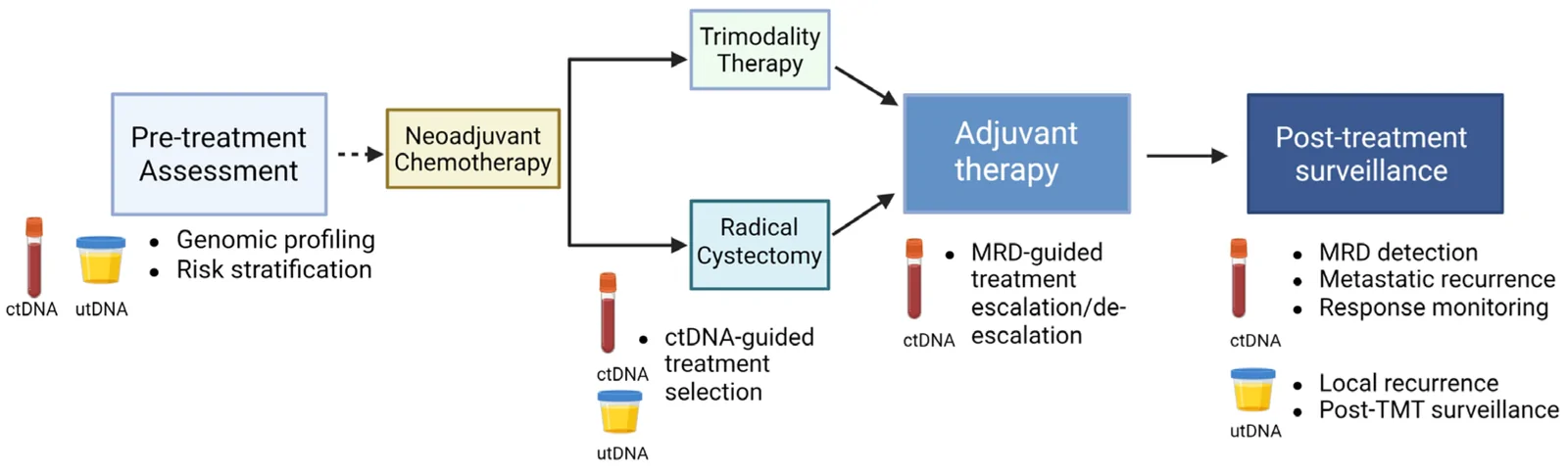
Potential clinical applications of ctDNA and utDNA throughout MIBC treatment. ctDNA and utDNA may complement clinical assessment by supporting pretreatment risk stratification, MRD-guided treatment selection, adjuvant therapy escalation or de-escalation, and surveillance for local recurrence and metastatic progression.

Beyond NMIBC, recent studies suggest that combined utDNA and ctDNA analysis may guide perioperative treatment decisions and prognostic stratification in MIBC (Figure 2). A recent analysis of the phase III NIAGARA trial evaluated utDNA and ctDNA in MIBC patients receiving neoadjuvant gemcitabine–cisplatin with or without perioperative durvalumab, followed by RC. Pre-RC utDNA clearance was associated with longer EFS, and pre-RC utDNA negativity was associated with pCR. In contrast, plasma ctDNA negativity was associated with longer EFS but not pCR, suggesting that ctDNA may better reflect systemic disease risk than pathological response at cystectomy [79]. Patients dual-negative for both biomarkers before RC had the most favorable outcomes, supporting the value of combining ctDNA and utDNA for prognostic stratification [79].

The combination of utDNA and ctDNA was also investigated in the phase II SunRISe-4 trial, which included MIBC patients who were either treated with neoadjuvant TAR-200 plus cetrelimab or cetrelimab monotherapy. Pre-RC utDNA negativity and clearance were strongly associated with pCR. In contrast, neither pre-RC ctDNA negativity nor ctDNA clearance was significantly associated with pCR, although ctDNA negativity at baseline and before RC was associated with longer RFS [80,81]. At baseline, utDNA was detectable in approximately 82% of patients regardless of whether TURBT was visibly complete or incomplete, supporting its potential to detect residual intravesical disease after apparently complete resection [80,81].

Findings from NIAGARA and SunRISe-4 differ from earlier neoadjuvant studies that reported significant associations between ctDNA-negativity and pathological response. For example, Dyrskjøt et al. associated pre-RC ctDNA negativity with pCR, and ABACUS linked ctDNA clearance to pCR [52,82]. However, interpretation is limited by the small relevant subgroups: only 10 patients were ctDNA-positive before RC in the Dyrskjøt analysis, and only 3 patients achieved ctDNA clearance in ABACUS [52,82]. In contrast, NIAGARA evaluated ctDNA in a substantially larger population of 265 biomarker-evaluable patients [79]. Moreover, SunRISe-4 evaluated intravesical gemcitabine-based therapy plus immunotherapy, a setting in which utDNA may more directly reflect local tumor response than plasma ctDNA [80,81]. Thus, differences in cohort size and treatment setting may contribute to these discordant results.

Taken together, these studies support a working model in which utDNA more closely reflects local bladder disease, whereas plasma ctDNA is generally more informative for systemic disease and metastatic recurrence risk. Plasma ctDNA is less sensitive in bladder-confined disease [58,66], whereas utDNA may be limited by field effects and preanalytical variability and may be less informative for systemic disease [74,76,79]. These differences are relative rather than absolute, because localized tumors can still shed ctDNA into plasma [29]. Nevertheless, complementary use of ctDNA and utDNA may improve disease assessment and risk stratification across bladder cancer stages.

## 9. Conclusions

Liquid biopsy approaches have rapidly expanded the potential for precision management of MIBC. Across neoadjuvant, adjuvant, and surveillance settings, ctDNA has demonstrated strong prognostic validity. Detectable ctDNA and failure to achieve clearance are consistently associated with higher recurrence and poorer survival [28,63]. IMvigor011 provides the clearest evidence of ctDNA’s clinical utility. The trial demonstrated that post-cystectomy ctDNA-positive patients with MIBC benefited from adjuvant atezolizumab, leading to its FDA approval in this setting [18,43,83]. Researchers are also investigating the utility of ctDNA in guiding other therapeutic strategies, including initiating immunotherapy at molecular relapse, selecting nivolumab-based adjuvant therapy, and de-escalating enfortumab vedotin plus pembrolizumab in advanced disease [65,84,85].

The selection of a liquid biopsy approach should be tailored to the disease setting and intended clinical use. Clinical evidence supports plasma ctDNA for assessing systemic MRD and stratifying metastatic recurrence risk, whereas utDNA may be better suited to evaluating residual intravesical disease and local treatment response [81]. utDNA-guided treatment de-escalation, repeat TURBT, and surveillance modification remain investigational [86]. In MIBC, integrating ctDNA and utDNA is particularly promising, as these complementary biomarkers may provide a more comprehensive assessment of systemic and local disease and ultimately support individualized treatment escalation or de-escalation strategies.

## 10. Future Outlook

Several technical and biological challenges must be addressed before ctDNA-based liquid biopsy can be routinely incorporated into clinical management. While highly sensitive methods such as digital droplet PCR and deep targeted sequencing can improve detection of low-abundance variants, implementation will require further standardization of assay design and validation methods across patient cohorts [87]. Additionally, patients with low-, non-shedding, or anatomically confined disease may experience false-negative ctDNA results [66]. Conversely, non-tumor-derived alterations, particularly those arising from clonal hematopoiesis, may generate false-positive signals, underscoring the need for matched normal sequencing and tumor-informed filtering strategies to distinguish tumor-derived variants from benign background mutations [88,89]. For Signatera™ CDx, assay failure may result from inadequate tumor content or DNA input during personalized assay development, insufficient plasma cfDNA, or failure to meet sequencing quality-control metrics [90].

The optimal timing and frequency of serial ctDNA testing also require standardization, as some studies evaluated ctDNA every 6 weeks, whereas others used variable testing intervals [58,83]. Differences in sampling intervals and assay-specific detection thresholds between platforms such as Signatera™ and personalized ddPCR assays hinder direct comparisons of ctDNA clearance across studies [63,65]. Discordant findings across studies further reflect limited cohort sizes and differences in treatment settings, including the reduced sensitivity of plasma ctDNA for assessing local treatment response in bladder-confined disease [29,52,79,80,81].

Practical and clinical barriers also remain. High cost and variability in reimbursement policies across Medicare and private insurers limit widespread access to ctDNA testing and hinder routine clinical implementation [91]. Finally, while persistently ctDNA-negative patients have favorable outcomes, robust randomized evidence is needed before ctDNA-guided omission of adjuvant therapy can be safely implemented [18,83].

Prospective studies continue to evaluate whether ctDNA-guided treatment adaptation can improve outcomes in MIBC (Table 1), particularly as novel perioperative regimens emerge for patients ineligible for or who decline cisplatin-based chemotherapy. The ongoing PRISMA-1 trial uses ctDNA to assess response to neoadjuvant ADC and ICI combinations and to identify patients for additional postoperative immunotherapy [92]. In addition, the MODERN trial directly tests ctDNA-guided adjuvant immunotherapy escalation and de-escalation after RC by randomizing ctDNA-positive patients to nivolumab with or without relatlimab, and ctDNA-negative patients to nivolumab or surveillance [84].

Emerging liquid biopsy approaches may extend beyond mutation-based ctDNA detection. Using urinary cfDNA fragmentation hotspots, Meng et al. developed a machine-learning classifier that distinguished bladder cancer from benign and healthy controls with 87% sensitivity at 100% specificity [93]. Similarly, Zhang et al. analyzed plasma cfDNA fragment size, end motifs, and breakpoint motifs using low-coverage whole-genome sequencing and machine learning, demonstrating strong discrimination between patients with bladder cancer and healthy controls [94]. Beyond fragmentomics, Zarbiv et al. prospectively evaluated a 21-locus urinary cfDNA methylation panel, demonstrating 90.5% sensitivity and 87.5% specificity for active bladder cancer [95]. A recent study used uRARE-seq, a sequencing method optimized for fragmented urine cell-free RNA (cfRNA), to detect localized bladder cancer with 95% sensitivity at 90% specificity. Pre-treatment urine cfRNA profiling identified gene expression signatures associated with response to intravesical therapy in NMIBC [96]. These approaches remain investigational and require prospective validation before clinical implementation, but they highlight the potential of multi-omic liquid biopsy strategies that integrate genomic, transcriptomic, fragmentomic, and methylation features with artificial intelligence-based data analysis.

Future liquid biopsy strategies may also include circulating tumor cells (CTCs), rare cancer cells shed from the primary tumor into the bloodstream [97]. CTCs provide multi-omic information and insight into tumor heterogeneity that ctDNA and utDNA cannot capture. Analyzing protein expression on the surface of CTCs may identify actionable targets, such as HER2, to guide targeted therapy [98,99]. CTC phenotyping may be especially useful in identifying therapeutic targets and biomarkers of treatment response as antibody–drug conjugates and immune checkpoint inhibitors are used more widely to treat MIBC [97,100].

In this era of rapid development in liquid biopsy, ctDNA and utDNA may offer complementary information on systemic and localized disease, respectively. Their integration with established histopathological assessment and imaging surveillance is poised to advance personalized treatment strategies in bladder cancer.

**Table 1:** Clinical studies evaluating ctDNA in muscle-invasive and metastatic bladder cancer.

Study/Trial [Ref]	Design	*N*	Context	Biomarker/ Assay	Prognostic/ Predictive	Key Findings	Limitations
ABACUS (NCT02662309) [52]	Prospective phase II, single-arm	95	MIBC; neoadjuvant atezolizumab	ctDNA; Signatera^TM^ (WES)	Prognostic	No relapses in baseline or post-neoadjuvant ctDNA− patients.	Exploratory biomarker analysis
RETAIN-1/2 (NCT04506554) [66]	Prospective phase II, single-arm	111	MIBC; neoadjuvant AMVAC ± nivolumab	ctDNA; Signatera^TM^ (WES)	Prognostic	Post-neoadjuvant ctDNA−: lower recurrence (34.8% vs 85.7%; *p* < 0.001).	Small ctDNA+ surveillance subgroup (*n* = 3)
Epstein et al. [58]	Prospective, observational	84	MIBC; TMT	ctDNA; Signatera^TM^ (WES)	Prognostic	Post-TMT ctDNA+: metastatic recurrence (*p* < 0.0001); no local association.	Clinical heterogeneity; variable testing intervals
Zhao et al. [59]	Retrospective, observational	32	MIBC; TMT	ctDNA; Signatera^TM^ (WES)	Prognostic	Post-TMT ctDNA+: metastatic progression (6-mo sensitivity 100%, specificity 93%).	Small cohort; variable testing intervals
CheckMate 274 [101]	Prospective phase III, RCT	709	MIBC; adjuvant nivolumab	ctDNA; Signatera^TM^ (WES)	Prognostic Predictive	Baseline ctDNA− vs. ctDNA+: DFS 52.1 vs. 5.0 mo (HR 0.30); nivolumab vs. placebo DFS HR 0.35 (ctDNA+) vs. 0.99 (ctDNA−).	Post hoc analysis; small ctDNA subset (133/709)
IMvigor010 (NCT02450331) [62,63]	Prospective phase III, RCT	809	MIBC; adjuvant atezolizumab	ctDNA; Signatera^TM^ (WES)	Prognostic Predictive	Baseline ctDNA+: worse OS (HR 6.3, 95% CI 4.45–8.92); atezolizumab improved DFS (HR 0.58, 95% CI 0.43–0.79).	Exploratory analysis; treatment not ctDNA-guided
IMvigor011 (NCT04660344) [18,83]	Prospective phase III, RCT	761	MIBC; adjuvant atezolizumab	ctDNA; Signatera^TM^ (WES)	Prognostic Predictive	Persistent ctDNA−: 1-/2-yr DFS 95%/88%; ctDNA+: atezolizumab improved DFS (HR 0.64) and OS (HR 0.59).	No ctDNA− treatment arm
TOMBOLA (NCT04138628) (2026) [65]	Prospective phase II, single-arm	192	MIBC; adjuvant atezolizumab	ctDNA; tumor-informed ddPCR (WES)	Prognostic	Post-RC ctDNA− vs. ctDNA+: 1-yr RFS 97% vs. 76%.	Incomplete follow-up; target accrual unmet
MODERN (NCT05987241) [84]	Prospective phase II/III, RCT	992 *	MIBC; adjuvant nivolumab ± relatlimab	ctDNA; Signatera^TM^ (WES)	Predictive	Evaluates post-RC ctDNA-guided adjuvant immunotherapy.	Results pending
Eraky et al. [56]	Prospective, observational	94	Bladder cancer; RC	ctDNA; Signatera^TM^ (WES)	Prognostic	Post-RC ctDNA+: recurrence detection (100% sensitivity, 91.8% specificity, 84.5% PPV, 100% NPV).	Small cohort; short follow-up
PRISMA-1 (NCT06133517) (2025) [92]	Prospective phase II, single-arm	70 *	MIBC; perioperative SG + ZIM + DOM	ctDNA; not reported	Predictive	Evaluates post-RC pCR- and ctDNA-guided adjuvant therapy.	Results pending
Sfakianos et al. [51]	Retrospective, observational	167	Bladder cancer; RC	ctDNA; Signatera^TM^ (WES)	Prognostic	Post-RC ctDNA+: shorter DFS in MRD (HR 6.93) and surveillance (HR 23.02) periods.	Small cohort; variable testing intervals
Christensen et al. (2019) [28]	Prospective, observational	68	MIBC; NAC	ctDNA; Signatera^TM^ (WES)	Prognostic	ctDNA+: shorter RFS pre-NAC (HR 29.1), pre-RC (HR 12.0), post-RC (HR 129.6).	Small cohort; short follow-up
Vandekerkhove et al. (2017) [27]	Prospective, observational	51	MIBC; platinum-based chemotherapy	ctDNA; tumor-naïve targeted NGS, WES in subset	N/A	ctDNA identified ERBB2 amplification & PIK3CA mutations (20% of patients each).	Small, heterogeneous cohort
Vandekerkhove et al. (2021) [23]	Retrospective, observational	104	mUC; systemic therapy	ctDNA; tumor-naïve NGS, WES in subset	Prognostic	ctDNA–tissue concordance 83.4%. Higher baseline ctDNA fraction: worse OS (HR 3.59, 95% CI 1.47–8.75).	Heterogeneous cohort; variable testing intervals
CT-READ (NCT06313666) (2026) [85]	Prospective phase II, single-arm	30 *	mUC; first-line EV + pembrolizumab	ctDNA; tumor-informed multiplex PCR	Prognostic Predictive	Tests ctDNA-guided EV discontinuation after 6 mo of combination therapy.	Small cohort; results pending
Christensen et al. (2023) [61]	Prospective, observational	92	MIBC; NAC	ctDNA and utDNA; tumor-informed NGS (Signatera™ /custom panels)	Prognostic	Pre-NAC ctDNA+: lower NAC response (*p* < 0.001). ctDNA/utDNA during NAC stratified RFS (*p* = 0.003).	Different ctDNA assays
NIAGARA (NCT03732677) [79]	Prospective phase III, RCT	1063	MIBC; NAC ± perioperative durvalumab	ctDNA and utDNA; Signatera^TM^ (WES)	Prognostic	Pre-RC ctDNA−/utDNA− vs. ctDNA+/utDNA+: 24-mo EFS 90% vs. 55%. Durvalumab increased utDNA clearance (39% vs. 27%).	Exploratory analysis; limited biomarker subset (265/1063)
SunRISe-4 [81]	Prospective phase II, RCT	163	MIBC; neoadjuvant cetrelimab ± TAR-200	ctDNA: Signatera™; utDNA: UroAmp™	Prognostic	utDNA− at week 12: higher pCR (*p* < 0.00001). ctDNA− at baseline/week 12: longer RFS (*p* = 0.04/0.01).	Exploratory analysis; short follow-up

Note: When reported, ctDNA was derived from plasma and utDNA from urine. Abbreviations: RCT = randomized controlled trial; SG = sacituzumab govitecan; ZIM = zimberelimab; DOM = domvanalimab; EV = enfortumab vedotin. * Planned enrollment.

## Data Availability

No new data were created or analyzed in this study. Data sharing is not applicable to this article.

## Abbreviations

The following abbreviations are used in this manuscript:

ctDNA	Circulating tumor DNA
utDNA	Urinary tumor DNA
cfDNA	Cell-free DNA
MRD	Molecular residual disease
MIBC	Muscle-invasive bladder cancer
mUC	Metastatic urothelial carcinoma
NMIBC	Non-muscle-invasive bladder cancer
RC	Radical cystectomy
TMT	Trimodality therapy
TURBT	Transurethral resection of bladder tumor
NAC	Neoadjuvant chemotherapy
pCR	Pathologic complete response
OS	Overall survival
DSS	Disease-specific survival
DFS	Disease-free survival
RFS	Recurrence-free survival
EFS	Event-free survival
NGS	Next-generation sequencing
WES	Whole-exome sequencing
WGS	Whole-genome sequencing
ddPCR	Digital droplet PCR
PCR	Polymerase chain reaction
SNV	Single-nucleotide variant
HTS	High-throughput sequencing
CT	Computed tomography
MRI	Magnetic resonance imaging
PET-CT	Positron emission tomography/computed tomography
BCG	Bacillus Calmette–Guérin
CTC	Circulating tumor cell
ADC	Antibody–drug conjugate
ICI	Immune checkpoint inhibitor
HR	Hazard ratio
CI	Confidence interval
